# CMV-independent increase in CD27−CD28+ CD8+ EMRA T cells is inversely related to mortality in octogenarians

**DOI:** 10.1038/s41514-019-0041-y

**Published:** 2020-01-21

**Authors:** Carmen Martin-Ruiz, Jedrzej Hoffmann, Evgeniya Shmeleva, Thomas von Zglinicki, Gavin Richardson, Lilia Draganova, Rachael Redgrave, Joanna Collerton, Helen Arthur, Bernard Keavney, Ioakim Spyridopoulos

**Affiliations:** 1 Biosciences Institute, Newcastle University, Newcastle upon Tyne, UK; 2 Institute of Neuroscience, Newcastle University, Newcastle upon Tyne, UK; 3 Department of Medicine, Cardiology, Goethe University Hospital Frankfurt, Frankfurt a. M., Germany; 4 Department of Pathology, University of Cambridge, Cambridge, UK; 5 Institute for Cell and Molecular Biosciences, Newcastle University, Newcastle upon Tyne, UK; 6 Translational and Clinical Research Institute, Newcastle University, Newcastle upon Tyne, UK; 7 UK Division of Cardiovascular Sciences, School of Medical Sciences, Faculty of Biology, Medicine and Health, The University of Manchester, Manchester, UK; 8 Manchester University NHS Foundation Trust, Manchester Academic Health Science Centre, Manchester, UK

**Keywords:** Biomarkers, Senescence

## Abstract

Cytomegalovirus (CMV) seropositivity in adults has been linked to increased cardiovascular disease burden. Phenotypically, CMV infection leads to an inflated CD8 T-lymphocyte compartment. We employed a 8-colour flow cytometric protocol to analyse circulating T cells in 597 octogenarians from the same birth cohort together with NT-proBNP measurements and followed all participants over 7 years. We found that, independent of CMV serostatus, a high number of CD27−CD28+ CD8 EMRA T-lymphocytes (TEMRA) protected from all-cause death after adjusting for known risk factors, such as heart failure, frailty or cancer (Hazard ratio 0.66 for highest vs lowest tertile; confidence interval 0.51–0.86). In addition, CD27−CD28+ CD8 EMRA T-lymphocytes protected from both, non-cardiovascular (hazard ratio 0.59) and cardiovascular death (hazard ratio 0.65). In aged mice treated with the senolytic navitoclax, in which we have previously shown a rejuvenated cardiac phenotype, CD8 effector memory cells are decreased, further indicating that alterations in T cell subpopulations are associated with cardiovascular ageing. Future studies are required to show whether targeting immunosenescence will lead to enhanced life- or healthspan.

## Introduction

We have previously shown the impact of cytomegalovirus (CMV) seropositivity, which reflects chronic latent infection with this herpes virus, on cardiovascular (CV) mortality in octogenarians.^1^ So far, no study has investigated the CMV-related T-cell phenotype in detail or its association with outcome in such a large population. Our goal was to assess the impact of CMV-related as well as unrelated changes to the T-cell phenotype to CV mortality in octogenarians, using a polychromatic, 8-colour protocol to accurately assess the T-cell phenotype by flow cytometry^2^ in 597 octogenarians from the same cohort (Newcastle 85+ study).^3,4^ In addition, patients were characterized more accurately regarding (a) pre-existing comorbidity, such as subclinical heart failure (reflected by increased NT-proBNP plasma levels) or frailty, (b) in-depth description of circulating T cell phenotypes and (c) 7 years follow-up data on health and mortality outcomes. We found in this unique cohort of octogenarians that among peripheral blood immune cells lymphocytes have the strongest predictive power for outcome. Among lymphocytes a large amount of cytotoxic CD8 T_EMRA_ cells as well as their loss of CD28 are independent predictors of increased mortality. It appears that virus-dependent as well as -independent changes to the CD8 T cell compartment are strong determinants of outcome in unselected octogenarians.

## Results and discussion

### Baseline parameters and gender differences

In 597 participants from phase II of the Newcastle 85+ study (86.5 years old, all born in 1921), male participants (38 %) were more likely to be smokers (67% vs 45%), have CV disease (CVD, 53% vs 43%), and previous myocardial infarction (20% vs 11%) than female participants (Supplementary Table 1). In contrast, males were less frail (11% vs 23%) than females. Despite similar CMV-seropositivity in both groups (84% vs 86%, Supplementary Table 1), women showed higher lymphocyte counts (1.7 vs 1.55 × 10^9^/μl, Supplementary Table 2), an increased CD4/CD8 ratio (2.7 vs 2.1), a higher frequency of naïve CD4 (47.2% vs 37.4%) and finally more naïve CD8 T-cells (8.5% vs 5.4%, Supplementary Table 3). This was unrelated to the higher prevalence of CVD (Supplementary Table 4).

### Cytomegalovirus seropositivity drives CD27–CD28− effector memory phenotype

We defined CD8 T_EMRA_ cells explicitly according to the commonly used Sallusto classification as CD8 T-lymphocytes lacking the C–C chemokine receptor type 7 (CCR7) and re-expressing CD45RA, thus CD3+CD4–CD8+CCR7−CD45RA+.^5^ The other T-lymphocyte subsets were classified as naïve (CCR7+CD45RA+), central memory (CM CCR7+CD45RA–) and effector memory (EM; CCR7−CD45RA−) cells. Of note, some authors chose a slightly different, less detailed subclassification for T-cell subsets by replacing CD45RA with CD27.^6^ CMV seropositivity in our cohort did explain (a) a lower CD4/CD8 ratio (2.2 vs 5.0) due to a higher proportion of CD8 T-cells, (b) reduced CD4 and CD8 naïve cells and (c) a higher proportion of CD8 T_EMRA_ cells (57% vs 32%, Supplementary Tables 5 and 6). The most significant difference in phenotype though was the very large increase in CD27/CD28 double negative subpopulations across all CD4 and CD8 memory T cell subsets (Supplementary Tables 5 and 6, Supplementary Fig. 1b). CMV-specific T cells are known to increase mainly in elderly CMV seropositive individuals (above the age of 65), displaying a CD27−CD28− effector memory phenotype,^7^ commonly considered a hallmark of immunosenescence. This is believed to contribute to the weakened immune status observed in the elderly. Accordingly, Derhovanessian et al. demonstrated that individuals from families enriched for longevity (with 30% lower mortality risk compared to the general population) are resistant to CMV-driven, age-associated reduction in naive T cells and accumulation of CD27−CD28− effector memory T cells.^8^

### NT-proBNP is a strong predictor of outcome in octogenerians

**Fig. 1:**
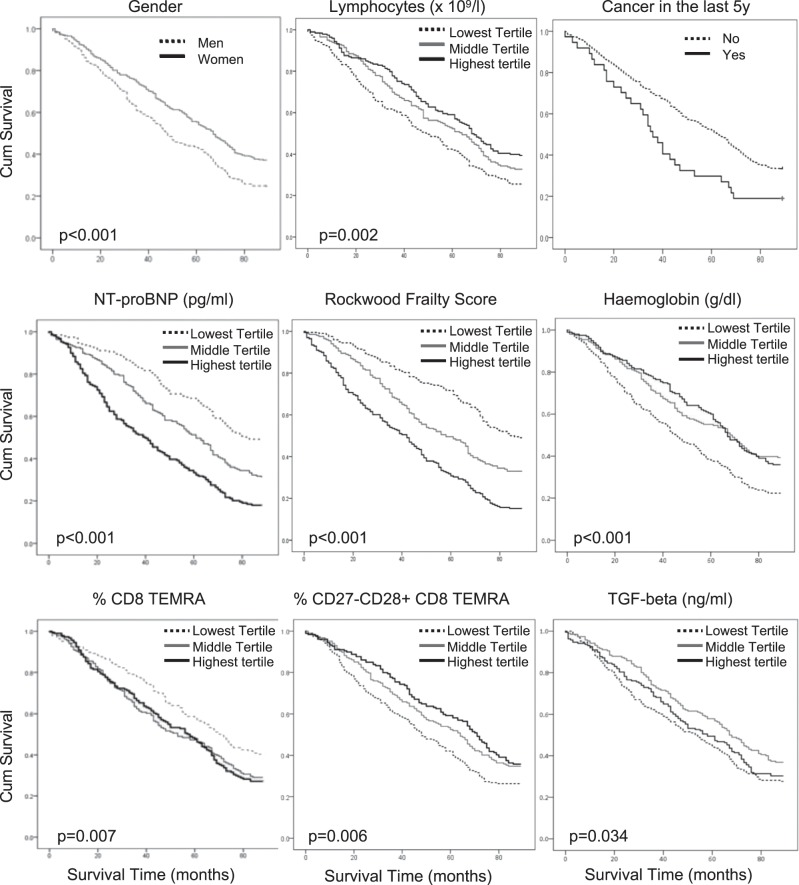
Gender, comorbidity and lymphocytes predict survival in the Newcastle 85+ study. Kaplan-Meier curves with their respective log-rank p-values identify male gender and pre-existing comorbidity, such as cancer, frailty, anaemia or heart failure (reflected by elevated plasma NT-proBNP) as predictors of adverse outcome in octogenarians. Likewise, low lymphocyte counts (tertiles <1420 × 10^6^/μl, 1420–1940 × 10^6^/μl and >1940 × 10^6^/μl) and a low percentage of CD27−CD28+ CD8+ T_EMRA_ cells (tertiles <1.9%, 1.9–3.4% and >3.4%) are singular predictors of reduced survival.

Plasma samples for NT-proBNP measurement were aliquoted on day of collection and stored at −80 °C. NT-proBNP was measured by an electrochemiluminescent sandwich immunoassay using the Modular Analytics E170 system (Roche Diagnostics, Lewes, UK). The between-batch coefficient of variation was 1.5–3.5% from 122–4322 ng/l, with an analytical range of 5–35000 ng/l. The use of NT-proBNP measurements makes part of clinical guidelines to reduce the number of unnecessary echocardiograms, as low levels of NT-proBNP rule out a diagnosis of heart failure.^9^ A recently published multi-centre study, within a combined in-patient and out-patient setting and a follow-up of 2 years, showed that NT-proBNP levels at clinical stabilisation are strongly and similarly related to survival in heart failure regardless of ejection fraction and that a given level of NT-proBNP portends the same risk of death in heart failure with preserved (HFpEF) or reduced (HFrEF) ejection fraction.^10^ The strong association between NT-proBNP and outcomes in HF regardless of EF is clinically important. NT-proBNP reflects LV wall stress, and is therefore expected to be lower in HFpEF than HFrEF on the basis of LaPlace’s Law. Yet, for a given level of NT-proBNP, prognosis for HFpEF was as poor as for HFrEF.^10^ NT-proBNP measurements thus provide a critical tool for clinicians to risk stratify their patients with HFpEF, whether in the stable situation or the acute decompensated state. As shown in Fig. 1, NT-proBNP plasma levels are a strong predictor of all-cause mortality in octogenarians (*p* < 0.001).

### Survival and Cox-regression analysis

**Fig. 2:**
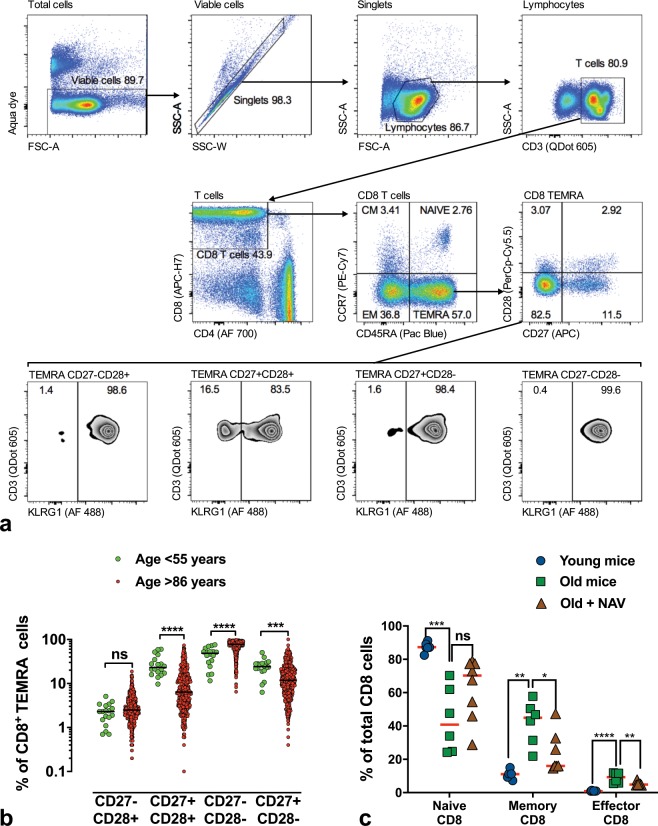
Loss of naïve CD8 T-cells with increased age in men and mice. **a** Gating strategy for CD8 T_EMRA_ subsets shows viable cells, singlets and lymphocyte gates, followed by T-cells (top row), CD8+ T-cells and further classification into naïve, CM, EM and T_EMRA_ CD8+ cells depending on their CCR7 and CD45^RA^ expression (middle row). The latter are then plotted against CD27 and CD28 co-receptors (middle row), and KLRG1 expression is depicted for each of the four CD27/CD28 subsets (lower row). **b** Distribution of CD8 T_EMRA_ subsets according to CD27 and CD28 co-receptors (healthy young control group (*n* = 18) vs. 85+ study population (*n* = 565). Loss of CD27/CD28 double positive cells with higher age. **c** Percentage of T-cell subsets from mouse splenocytes, relative to total CD8 population, in different experimental groups (young *n* = 6, old *n* = 7, and old mice treated with navitoclax *n* = 8). Data are depicted as single data points and mean (red line). ***p < 0.001; **p < 0.01; *p < 0.05 using 1-way ANOVA.

Independent determinants for all-cause mortality were as expected: gender, frailty, pre-existing heart failure (reflected by NT-proBNP) and cancer (Fig. 1). Interestingly, higher mortality in men vs women (75% vs 63%, *p* = 0.002) was exclusively related to increased CV mortality (30.2 vs 21.8%, Supplementary Table 1). Following adjustment of the Cox-regression analysis for heart failure (NT-proBNP), cancer and frailty, the only independent predictors remaining were total lymphocyte count reflecting thymic function (hazard ratio (HR) and 95% confidence interval (CI) for highest vs. lowest tertile = 0.70 and 0.53–0.93 respectively), percentage of CD8 T_EMRA_ cells (HR 1.46; CI 1.12–1.92) as a surrogate for immunosenescence, and finally expression of the co-receptor CD28 in CD8 T_EMRA_ cells (HR 0.66; CI 0.51–0.86) (Tables 1 and 2). Non-cardiovascular mortality was predicted by a low hemoglobin and T_EMRA_ cells as well as expression of CD28 (Supplementary Table 7). Cardiovascular mortality was predicted by high basophil number (reflecting inflammation), low levels of plasma TGF-beta and lack of CD28 expression in the CD27− CD8 + T_EMRA_ cells (Supplementary Table 7). While the main three subsets of CD8 EMRA T-cells were all age and CMV-dependent, thus all linked to immunosenescence, CD27−CD28 + CD8 + T_EMRA_ cells were not (Fig. 2a, b). This sub-population ranged between 0.2% and 20% of the whole EMRA population (Fig. 2b) and demonstrated high KLRG1 levels (Fig. 2a, Supplementary Fig. 1C), which is preferentially found on antigen-experienced CD8 memory T-cells (Supplementary Fig. 1A). Virus-specific CD8+ T cells are mostly KLRG1+ in chronic human viral infections, such as CMV, EBV and HIV, but not in resolved infection (e.g. influenza virus).^11^ Verma et al. have also shown recently that the CD27−CD28+ subset of the heterogenous CD8 T_EMRA_ population does not express the differentiation marker CD57, has longer telomeres than the CD28− subsets, and is capable of high proliferative capacity and differentiation plasticity,^12^ hence the expression of CD28 in CD27− CD8 T cells likely reflects a subset of non-terminally differentiated effector cells.

**Table 1:** All cause mortality Cox-regression model: CD8 TEMRA.

		N° Individuals	N° Events	Unadjusted hazard ratio	*p*	Adjusted hazard ratio^a^	*p*
Lymphocytes (×10^9^/l) Tertiles	≤1.42	196	146	1	0.006	1	0.046
	1.43–1.93	199	134	0.76 (0.59–0.99)		0.84 (0.64–1.09)	
	1.94+	193	117	0.64 (0.49–0.85)		0.70 (0.53–0.93)	
Basophils (×10^9^/l) Tertiles	≤0.03	281	183	1	0.162	1	0.134
	0.04–0.05	180	126	1.28 (0.99–1.64)		1.30 (1.01–1.67)	
	0.06+	127	88	1.13 (0.85–1.51)		1.13 (0.84–1.50)	
TGF-beta (ng/ml) Tertiles	≤13.17	188	136	1	0.033	1	0.227
	13.18–17.36	190	120	0.72 (0.55–0.94)		0.82 (0.62–1.06)	
	17.37+	188	131	0.94 (0.72–1.23)		1.00 (0.76–1.31)	
Haemoglobin (g/dl) Tertiles	≤12.30	197	153	1	0.001	1	0.112
	12.31–13.60	196	119	0.68 (0.53–0.88)		0.79 (0.61–1.02)	
	13.61+	195	125	0.66 (0.51–0.85)		0.79 (0.61–1.03)	
CD8 TEMRA (%) Tertiles	≤45.20	186	111	1	0.006	1	0.014
	45.21–63.80	186	132	1.36 (1.04–1.77)		1.37 (1.05–1.80)	
	63.81+	184	134	1.53 (1.17–1.99)		1.46 (1.12–1.92)	

Values are expressed as Mean (Lower, Upper bound of 95% CI)

^a^Adjusted for Tertiles of NT-proBNP, Cancer and Frailty

**Table 2:** All cause mortality Cox-regression model: CD8 CD27–CD28+.

		N° Individuals	N° Events	Unadjusted hazard ratio	*p*	Adjusted hazard ratio^a^	*p*
Lymphocytes (x10^9^/l) Tertiles	≤1.42	196	146	1	0.068	1	0.200
	1.43**–**1.93	199	134	0.84 (0.65**–**1.09)		0.94 (0.72**–**1.22)	
	1.94+	193	117	0.72 (0.55**–**0.95)		0.78 (0.59**–**1.03)	
Basophils (x10^9^/l) Tertiles	≤0.03	281	183	1	0.138	1	0.099
	0.04**–**0.05	180	126	1.28 (1.00**–**1.65)		1.32 (1.02**–**1.70)	
	0.06+	127	88	1.18 (0.88**–**1.57)		1.18 (0.88**–**1.57)	
TGF-beta (ng/ml) Tertiles	≤13.17	188	136	1	0.038	1	0.281
	13.18**–**17.36	190	120	0.72 (0.55**–**0.94)		0.82 (0.63**–**1.06)	
	17.37+	188	131	0.92 (0.70**–**1.20)		0.96 (0.74**–**1.26)	
Haemoglobin (g/dl) Tertiles	≤12.30	197	153	1	0.000	1	0.072
	12.31**–**13.60	196	119	0.65 (0.50**–**0.84)		0.77 (0.59**–**1.00)	
	13.61+	195	125	0.63 (0.49**–**0.82)		0.77 (0.59**–**1.01)	
CD8 CD27–CD28+ (%) Tertiles	≤1.90	190	140	1	0.013	1	0.004
	1.91**–**3.40	187	122	0.78 (0.60**–**1.00)		0.72 (0.55**–**0.93)	
	3.41+	179	115	0.68 (0.53**–**0.89)		0.66 (0.51**–**0.86)	

Values are expressed as Mean (Lower, Upper bound of 95% CI)

^a^Adjusted for Tertiles of NT-proBNP, Cancer and Frailty

### Senolytic therapy

CD8+ T_EMRA_ cells have been shown to be functionally senescent, as demonstrated by both proliferative arrest and the increased production and secretion of inflammatory mediators characteristic of a senescence-associated secretory phenotype (SASP).^6^ In proof of principle experiments we therefore aimed to investigate whether it was possible to reduce immunosenescence in aged mice using a senolytic drug, which we have previously shown attenuates the age-related cardiac phenotype.^13,14^ Indicative of immunosenescence, CD8 effector memory cells were increased and naïve CD8 T-cells were significantly decreased in aged mice. This phenotype was rescued by navitoclax (Fig. 2c). While in the current study we have not investigated the mechanisms by which senolytics increase the naive T-cell population, clearance of senescent cells via senolytics promotes hematopoietic stem cell function^15^ and in human trials, senolytic treatment attenuates circulating SASP.^16^ It is, therefore, possible that as well as eliminating senescent CD8 cells senolytic treatment influences the balance of different T-cell subpopulations via changing the dynamics of lymphocyte proliferation as a result of reduced systemic inflammation or rejuvenation of progenitor pools.

In conclusion, out of all immune cells, the subset of CD27− CD28+ CD8+ T_EMRA_ cells appeared to be the strongest independent predictor of all-cause mortality in the elderly. Most likely, this subset reflects the size of a T_EMRA_ subcompartment with retained plasticity and proliferative capacity, among otherwise senescent effector memory cells. Therefore, they might represent quickly accessible effector memory cells in the elderly, able to combat infections. Alternatively, a large pool of CD28+ cells indicates that less terminally differentiated EMRA cells are necessary to control chronic latent infections such as CMV, hence they are indicative of less underlying subclinical inflammation. Future studies have to show whether targeting immunosenescence and/or increasing immunocompetent immune cells with senolytics, or by telomerase-activators such as TA-65,^17,18^ will lead to enhanced life- or healthspan. Our TACTIC trial (Telomerase ACTivator to reverse Immunosenescence in Acute Coronary Syndrome: a double-blind, phase II, Pilot randomized controlled trial; ISRCTN 16613292) will show whether activation of telomerase by 12-month treatment with TA-65 in patients over the age of 65 with acute coronary syndrome reduces the percentage of pro-inflammatory CD8 T_EMRA_ lymphocytes, increases telomere length, and finally antagonize systemic inflammation and SASP.

## Methods

### Study population

The recruitment strategy for the Newcastle 85+ Study has been previously published.^3,4^ Briefly, individuals born in 1921 living in Newcastle or North Tyneside (North-East England) were recruited at around age 85 through their general practice, including those in institutional accommodation and those cognitive impaired. The Newcastle 85+ cohort is socio-demographically representative of the North-East of England as well as the population of England and Wales.^4^ The study conformed to the requirements of the Declaration of Helsinki and received ethical approval from the Newcastle and North Tyneside 1 Research Ethics Committee (reference number 06/Q0905/2). We sought written informed consent from participants directly or, when they lacked such capacity to consent, from a relative or carer.

### PBMCs isolation, cell cryopreservation and storage

Peripheral blood mononuclear cells (PBMCs) were obtained after Ficoll density gradient centrifugation using Ficoll-Hypaque. Following isolation, PBMCs were carefully resuspended in ice-chilled freezing medium containing RPMI 1640 with 10% FBS, 1% P/S and 10% DMSO (1 ml medium per 1 × 10^6^ cells) and aliquoted into cryovials. Cell aliquots were frozen at −80 °C and stored in a liquid nitrogen tank until use.

### Lymphocyte immunophenotyping

Blood samples were analysed by 8-colour flow-cytometry assays, which were performed similarly as previously described.^2^ Shortly, frozen PBMC aliquots were quickly thawed, and washed using an automatic cell washer. Following primary wash step, cell viability and cell numbers were analyzed using Vi cell counter. PBMC staining was performed with CD3-QDot605 and CD45RA-PacificBlue (both Invitrogen), CD4-AlexaFluor700, CD8-APC-H7, CCR7-PE/Cy7, CD27-APC, and CD28-PerCp/Cy5.5 (all BD Biosciences). Following three wash steps, LIVE/DEAD® Fixable Aqua Dead Cell Stain (Thermo Fisher Scientific, Waltham, MA, USA) was added to allow discrimination of viable and dead cells. Samples were measured on a BD LSR II cytometer using BD FACSDiva acquisition software. At least 100,000 viable cell events per sample were acquired. The gating scheme is depicted in Fig. 2a.

### Animals, procedures and senolytic treatment

C57BL/6 mice were analyzed at either 13 weeks (3 months) or 100 weeks (23 months) of age. Mice were purchased from Charles River (Charles River Laboratories International, UK). The project was approved by the Faculty of Medical Sciences Ethical Review Committee, Newcastle University (project license no. 60/3864). At ~23 months of age, mice were randomly assigned to a treatment group. ABT263 (navitoclax) or vehicle alone was administered to mice by gavage at 50 mg/kg body weight per day (mg/kg/d) for 7 day per cycle for two cycles with a 1-week interval between the cycles. Mice from each treatment group were then culled by humane methods and their spleen collected.

### Mouse flow-cytometry

Dissected spleens were ground on a pre-wet 70 μm filter with a 2 ml syringe plunger. The filters were washed with 10 ml PBS and cell suspension centrifuged at 300 × *g* for 5 min at 4 °C. The pellet was resuspended in 3 ml Pharm Lyse red blood cell lysis buffer (BD) and incubated for 5 min at room temperature. Ten ml Flow-cytometry buffer (PBS + 1% BSA + 0.05% NaN3) was used to stop the reaction, followed by another centrifugation step. Cell suspension was filtered through a 30 μm filter and cells were counted using Tali chips (Thermo Fisher Scientific). Fc receptors of cells were blocked using 1:50 anti-CD16/32 (clone 93, BioLegend). Around 1 × 10^6^ cells were stained with BV605 anti-CD3 (clone 17A2, BioLegend), BV480 anti-CD4 (clone RM4-5, BD), PerCP-Cy5.5 anti-CD8 (clone 53–6.7, BioLegend), BUV395 anti-CD44 (clone IM7, BD) and PE anti-CD62L (clone MEL-14, BioLegend). The cells were incubated for 60 min on ice and washed twice with staining buffer. A 1:10 DAPI solution was used to distinguish live and dead cells. Flow-cytometry data were acquired on a BD Fortessa analyser and analysed using FCS Express (V6, De Novo Software). Please see gating scheme in Supplementary Fig. 2.

### Blood-based biomarkers

Blood samples were collected within 6 months of participant assessment. Biomarkers were measured from a fasting blood sample that reached the lab within 1 h for processing. A comprehensive list of the biomarkers included is in Supplementary Table 2 and the methodological details of the assays can be found in refs. ^1,19,20^

### Morbidity, mortality and causes of death

A multidimensional health assessment was carried out during three visits in the participant’s usual dwelling by a dedicated research nurse. Data on pre-existing diseases and prescribed medication were obtained from reviewing the medical records held for the participants at their respective general practice. Date and cause of death were obtained through the UK Health and Social Care Information Service. Survival time (in months) was calculated from date of blood draw to date of death or censored at 30 April 2015 (median follow-up 61 months, overall survival rate 32.5%). We defined cardiovascular death as ICD codes I00–I69 and death due to myocardial infarction or stroke to be I20–I25 and I60–I69.

### Statistics

Our total cohort for the study comprised of 597 participants. We compared by gender the health measurements, socio-demographic and biomarkers distributions for our participants by Chi-square tests for categorical variables, with data represented as percentage, or by Mann-Whitney U tests for ordinal variables, with data represented as median and interquartile range (IQR) (Supplementary Tables 1 and 2). For each of those variables we performed a Kaplan-Meyer Survival analysis by categories or by tertiles (on ordinal variables) with a Log-rank test. The rational for the selection of covariates included on the final Cox-Regression Models was as follows:

- Forward selection Cox-Regression analysis was performed on tertiles of NT-proBNP and tertiles of Immunosenescence variables: subpopulations Naïve, CM, EM and TEMRA for CD4 and CD8, with an alternative model where CD8 TEMRA were replaced by detailed CD8 TEMRA CD27/CD28 subpopulations. Those models were performed adjusting for Gender, Anemia, Cancer, Diabetes, Renal Impairment, Tertiles of Rockwood Frailty Score and tertiles of Total cholesterol.
- Forward selection Cox-Regression analysis was performed on tertiles of NT-proBNP and tertiles of all remaining blood biomarkers. An alternative model was run with only those biomarkers with significant Log-rank Kaplan-Meyer survival analysis. Those models were performed adjusting, as before, for Gender, Anemia, Cancer, Diabetes, Renal Impairment, Tertiles of Rockwood Frailty Score and tertiles of Total cholesterol.

From the outcomes of the above we were able to select the 5 variables that we carried over to the final analysis:

Lymphocytes, Basophils, TGF-beta, Haemoglobin and either CD8 TEMRA or CD8 TEMRA CD27−CD28+, with the relevant adjustment for tertiles of NT-proBNP, Cancer and tertiles of Rockwood Frailty Score. The Cox-regression analysis were performed on overall Mortality/Survival as well as discriminating between Cardiovascular Mortality and Non-cardiovascular mortality. See Flow chart attached (Supplementary Fig. 3).

### Reporting summary

Further information on research design is available in the Nature Research Reporting Summary linked to this article.

## Supplementary information

Supplementary Material

Reporting Summary

## Data Availability

The datasets generated during and/or analysed during the current study are available from the corresponding author on reasonable request.
