# Supplementary material for: CMV-independent increase in CD27−CD28+ CD8+ EMRA T cells is inversely related to mortality in octogenarians
Source: NPJ Aging Mech Dis. 2020 Jan 21;6:3. doi: 10.1038/s41514-019-0041-y (PMC6972903; doi:10.1038/s41514-019-0041-y)

Martin-Ruiz et al.: Online Supplementary Information

Supplementary Tables

Supplementary Tables 1. Baseline characteristics by gender

Supplementary Tables 2. Biomarkers by gender

Supplementary Tables 3. 8-colour results by gender

Supplementary Tables 4. 8-colour results by previous MI

Supplementary Tables 5. 8-colour results by CMV

Supplementary Tables 6. 8-colour results by CMV and gender

Supplementary Tables 7. Cox Regression Analysis for cardiovascular and non-cardiovascular mortality

Supplementary Figures

Supplementary Figures 1. Phenotype of CD8 T cells in young (<55 years) and elderly (>86 years) participants.

Supplementary Figure 2: Mouse flow-cytometry gating scheme

Supplementary Figures 3. Flowchart on covariates selection.

Supplementary Table 1. Baseline Characteristics and Gender

GENDER	Male	Female		
	N=225	N=372	p value	
BMI (median, [IQR])	24.6 [22.36, 27.33]	24.2 [21.51, 27.33]		
CMV POSITIVE (%)	84.4%	86.0%	0.597	†
Smoking (%)				
Never	27.7%	42.0%		
Current	2.7%	6.2%	**0.000**	†
Former - Regular	66.5%	45.0%		
Former - Occasional	3.1%	6.7%		
Institutional Housing (%)	5.8%	9.5%	**0.039**	†
Education (more than 11y) (%)	15.2%	13.9%	0.819	†
CVD (%)	56.4%	46.0%	**0.013**	†
CVD - Atherosclerotic disease (%) [1]	53.3%	42.9%	**0.013**	†
CVD - Cerebrovascular Disease (%) [1]	21.8%	17.8%	0.232	†
CVD - Peripheral Vascular Disease (%) [1]	8.4%	5.4%	0.144	†
CVD - Heart Failure (%) [1]	9.8%	9.2%	0.804	†
CHD (%)	35.6%	29.6%	0.134	†
CHD - Angina (%) [1]	33.3%	27.5%	0.130	†
CHD - MI (%) [1]	20.0%	11.1%	**0.003**	†
Hypertension (%)	55.1%	59.3%	0.316	†
Diabetes (%)	13.3%	12.4%	0.740	†
Anemia (WHO's Guideline) (%)	29.5%	23.6%	0.122	†
Rheumatoid Arthritis (%)	0.4%	2.4%	0.068	†
Cancer, any (%)	26.2%	21.3%	0.167	†
Cancer, any (Excluding B&S,<5 years since diagnosis) (%)	7.6%	5.4%	0.288	†
Renal Impairment CKD-Epi (%)	23.4%	23.7%	0.944	†
Use of beta-blockers (%)	28.0%	27.8%	0.950	†
Frailty by Fried Index (%)	11.2%	22.9%	**0.000**	†
Rockwood frailty index (median, [IQR]) [a]	0.17 [0.11, 0.23]	0.2 [0.13, 0.28]	**0.000**	*
Survival (%) May 2015	24.9%	37.1%	**0.002**	*
Cause Mortality (%)				
Death from MI or Stroke [N]	30.2% [68]	21.8% [81]	**0.004**	†
Death from other than MI or Stroke [N]	44.9% [101]	41.1% [153]		
Follow-up time (in months) (median, [IQR]) May2015	48 [25, 83]	67 [35, 89]	**<0.001**	*

* Mann-Whitney; † Chi-Square; [1] Numbers refer to % of total population and some patients may have multiple entries.
CHD=Coronary Heart disease= Myocardial Infarction, Angina, Coronary Artery Bypass Graft or Coronary Angioplasty / Stent
CVD= Cardiovascular disease= Cerebrovascular disease, Peripheral vascular disease, Heart Failure and Atherosclerotic Disease.

Supplementary Table 2. Biomarkers by Gender

GENDER	Male<br>N=225	Female<br>N=372	p value	
Haematological markers				
Red blood cell count (x 10^{12}/l) (median, [IQR])	4.39 [4.06, 4.75]	4.27 [3.94, 4.57]	**0.001**	*
White blood cell count (x 10^{9}/l) (median, [IQR])	6.40 [5.40, 7.50]	6.60 [5.50, 7.90]	0.124	*
Platelets (x 10^{9}/l) (median, [IQR])	231.00 [195.00, 272.50]	265.00 [226.00, 311.00]	**0.000**	*
Neutrophils (x 10^{9}/l) (median, [IQR])	3.76 [3.08, 4.73]	3.94 [3.23, 4.98]	0.183	*
Monocytes (x 10^{9}/l) (median, [IQR])	0.54 [0.43, 0.66]	0.50 [0.41, 0.63]	0.054	*
Lymphocytes (x 10^{9}/l) (median, [IQR])	1.55 [1.23, 1.98]	1.70 [1.35, 2.16]	**0.003**	*
Haemoglobin (g/dl) (median, [IQR])	13.40 [12.40, 14.50]	12.80 [11.80, 13.70]	**0.000**	*
Basophils (x 10^{9}/l) (median, [IQR])	0.04 [0.02, 0.06]	0.04 [0.02, 0.05]	0.685	*
Eosinophils (x 10^{9}/l) (median, [IQR])	0.22 [0.15, 0.33]	0.19 [0.11, 0.28]	**0.001**	*
Metabolic Markers				
Total Cholesterol (mmol/l) (median, [IQR])	4.50 [3.80, 5.20]	5.20 [4.30, 6.00]	**0.000**	*
Triglycerides (mmol/l) (median, [IQR])	1.10 [0.90, 1.50]	1.40 [1.00, 1.80]	**0.000**	*
High Density Lipoprotein (HDL) (mmol/l) (median, [IQR])	1.30 [1.10, 1.60]	1.50 [1.30, 1.85]	**0.000**	*
Low Density Lipoprotein (LDL) (mmol/l) (median, [IQR])	2.50 [1.80, 3.10]	2.80 [2.10, 3.70]	**0.000**	*
Cholesterol/HDL Ratio (median, [IQR])	3.30 [2.70, 3.90]	3.20 [2.70, 3.90]	0.952	*
HbA1C (%) (median, [IQR])	5.90 [5.60, 6.10]	5.80 [5.60, 6.20]	0.442	*
Natriuretic Markers				
proBNP (pg/ml) (median, [IQR])	453.00 [231.00, 1306.00]	396.00 [178.00, 748.00]	**0.034**	*
Inflammatory and senescence-related Markers				
Leptin (ng/ml) (median, [IQR])	12.65 [7.61, 23.89]	27.00 [13.75, 52.09]	**0.000**	*
Adiponectin (ug/ml) (median, [IQR])	7.66 [4.84, 11.44]	9.05 [6.87, 13.13]	**0.000**	*
ApoE4 carrier (%)	24.2%	26.8%	0.522	†
Mitochondrial haplogroup J (%)	13.2%	10.5%	0.831	†
hsCRP (mg/l) (median, [IQR])	3.00 [1.20, 6.40]	2.60 [1.10, 5.80]	0.288	*
LPS-estimulated IL-6 (ng/ml) (median, [IQR])	10.15 [4.48, 16.49]	8.30 [4.89, 16.24]	0.243	*
LPS-estimulated TNF alpha (pg/ml) (median, [IQR])	431.52 [208.69, 858.33]	381.74 [179.27, 678.91]	**0.041**	*
IGFBP1 (ng/ml) (median, [IQR])	61.49 [35.67, 101.97]	62.40 [37.68, 101.91]	0.889	*
IGFBP3 (ng/ml) (median, [IQR])	712.61 [568.83, 833.08]	886.95 [760.90, 999.85]	**0.000**	*
TGFbeta (ng/ml) (median, [IQR])	14.07 [11.03, 17.53]	15.68 [12.81, 19.24]	**0.000**	*
Telomere length (kbp) (median, [IQR])	3.80 [3.56, 4.00]	3.67 [3.44, 3.91]	**0.001**	*
DNA Damage (%) (median, [IQR])	45.27 [28.80, 64.32]	46.33 [29.88, 62.52]	0.959	*
DNA Repair (%) (median, [IQR])	41.43 [20.75, 62.95]	44.89 [22.97, 69.39]	0.309	*
8-iso Prostaglandin F2alpha (ng/ml) (median, [IQR])	1.18 [0.67, 1.69]	1.22 [0.75, 2.56]	0.247	*
Telomerase activity (AU) (median, [IQR])	0.90 [0.60, 1.46]	1.03 [0.66, 1.59]	0.161	*

* Mann-Whitney; † Chi-Square

Supplementary Table 3. Immunosenescence Parametres by Gender

GENDER	Male<br>N=225<br>***(%) Median [IQR]***	Female<br>N=372<br>***(%) Median [IQR]***	***p-value<0.05***
CD3+	70.50 [63.60, 78.00]	72.50 [64.40, 79.40]	*
CD4	60.20 [47.60, 71.80]	65.60 [54.70, 77.20]	*
CD4 CD27-CD28-	4.90 [0.90, 12.40]	3.20 [0.80, 9.70]	*
CD4 CD27-CD28+	5.70 [3.40, 9.90]	4.50 [2.50, 7.60]	*
CD4 CD27+CD28-	0.50 [0.30, 0.90]	0.60 [0.30, 1.00]	
CD4 CD27+CD28+	87.80 [75.30, 93.70]	90.20 [81.50, 94.60]	*
CD4 CM	36.10 [27.90, 43.70]	31.50 [23.20, 40.50]	*
CD4 CM CD27-CD28-	0.00 [0.00, 0.10]	0.00 [0.00, 0.10]	
CD4 CM CD27-CD28+	3.60 [2.40, 5.60]	3.20 [2.10, 4.60]	
CD4 CM CD27+CD28-	0.10 [0.00, 0.30]	0.10 [0.10, 0.30]	
CD4 CM CD27+CD28+	96.00 [93.90, 97.00]	96.40 [94.90, 97.30]	
CD4 EM	16.30 [10.30, 26.40]	13.10 [8.50, 21.70]	*
CD4 EM CD27-CD28-	16.90 [4.90, 39.60]	16.70 [3.90, 35.70]	
CD4 EM CD27-CD28+	23.90 [18.30, 30.30]	21.00 [16.40, 27.70]	*
CD4 EM CD27+CD28-	0.40 [0.20, 0.60]	0.50 [0.20, 0.80]	
CD4 EM CD27+CD28+	51.30 [33.90, 68.30]	55.90 [39.80, 72.40]	*
CD4 NAIVE	37.40 [22.80, 48.80]	47.20 [33.50, 58.80]	*
CD4 NAIVE CD27-CD28-	0.00 [0.00, 0.10]	0.00 [0.00, 0.00]	
CD4 NAIVE CD27-CD28+	0.10 [0.00, 0.20]	0.00 [0.00, 0.10]	
CD4 NAIVE CD27+CD28-	0.40 [0.20, 0.80]	0.40 [0.20, 0.80]	
CD4 NAIVE CD27+CD28+	99.40 [98.80, 99.70]	99.40 [98.90, 99.70]	
CD4 TEMRA	1.40 [0.70, 3.60]	1.30 [0.70, 3.60]	
CD4 TEMRA CD27-CD28-	38.15 [8.90, 72.20]	38.80 [10.50, 68.80]	
CD4 TEMRA CD27-CD28+	10.85 [6.60, 18.00]	9.90 [5.40, 15.80]	
CD4 TEMRA CD27+CD28-	1.20 [0.35, 2.75]	1.50 [0.60, 3.00]	
CD4 TEMRA CD27+CD28+	43.40 [12.05, 70.95]	41.70 [17.70, 73.20]	
CD8+	30.10 [20.60, 43.20]	24.80 [16.20, 36.40]	*
CD8 CD27-CD28-	57.40 [32.60, 70.40]	51.70 [31.00, 66.70]	
CD8 CD27-CD28+	4.30 [2.80, 6.00]	3.50 [2.40, 5.00]	*
CD8 CD27+CD28-	9.00 [5.50, 15.10]	8.90 [5.90, 14.40]	
CD8 CD27+CD28+	27.00 [16.10, 46.30]	32.60 [20.60, 52.10]	*
CD8 CD45RA-CD27-	9.20 [5.10, 17.20]	6.70 [3.80, 11.80]	
CD8 CD45RA-CD27+	16.40 [9.50, 28.20]	17.60 [9.90, 28.40]	
CD8 CD45RA+CD27-	47.10 [29.00, 60.70]	44.50 [25.80, 59.90]	
CD8 CD45RA+CD27+	18.30 [11.40, 31.50]	23.70 [15.20, 37.80]	*
CD8 CM	6.30 [3.50, 12.30]	7.50 [4.20, 13.00]	
CD8 CM CD27-CD28-	0.70 [0.10, 1.90]	0.40 [0.10, 1.50]	
CD8 CM CD27-CD28+	5.80 [3.55, 8.35]	4.30 [2.50, 6.40]	*
CD8 CM CD27+CD28-	0.90 [0.30, 2.15]	1.10 [0.40, 1.90]	
CD8 CM CD27+CD28+	91.70 [87.25, 93.95]	93.50 [89.80, 95.60]	*
CD8 EM	21.80 [15.10, 31.70]	18.90 [12.00, 25.70]	*
CD8 EM CD27-CD28-	32.40 [12.10, 57.00]	28.60 [12.50, 49.50]	
CD8 EM CD27-CD28+	8.10 [5.10, 11.70]	7.00 [5.00, 10.70]	
CD8 EM CD27+CD28-	8.10 [4.90, 14.50]	9.10 [5.70, 14.30]	
CD8 EM CD27+CD28+	45.10 [24.00, 60.10]	48.30 [31.20, 64.50]	*
CD8 NAIVE	5.40 [2.10, 10.80]	8.50 [4.60, 18.00]	*
CD8 NAIVE CD27-CD28-	1.10 [0.30, 3.80]	0.80 [0.30, 2.30]	
CD8 NAIVE CD27-CD28+	0.20 [0.00, 0.70]	0.10 [0.00, 0.50]	
CD8 NAIVE CD27+CD28-	5.20 [3.20, 9.30]	5.10 [2.90, 7.80]	*
CD8 NAIVE CD27+CD28+	91.10 [86.20, 95.20]	92.90 [87.80, 95.50]	
CD8 TEMRA	54.90 [40.90, 68.10]	54.20 [39.00, 68.60]	
CD8 TEMRA CD27-CD28-	78.40 [59.00, 87.60]	76.40 [61.20, 85.40]	
CD8 TEMRA CD27-CD28+	2.50 [1.70, 3.80]	2.50 [1.60, 4.10]	
CD8 TEMRA CD27+CD28-	11.70 [5.90, 20.60]	12.00 [6.70, 20.30]	
CD8 TEMRA CD27+CD28+	5.80 [2.90, 12.40]	6.70 [3.50, 14.00]	
CD4/CD8 RATIO	2.07 [1.08, 3.42]	2.72 [1.53, 4.63]	*
CD3+ CD4- CD8-	4.40 [2.60, 7.90]	4.30 [2.50, 7.30]	
CD3+ CD4+ CD8+	0.60 [0.30, 1.20]	0.80 [0.50, 1.50]	*

Supplementary Table 4. Immunosenescence Parametres by MI

	No Myocardial Infarction *N=510*	Myocardial Infarction *N=86*	***Mann-Whitney test***
	(%) Median [IQR]	***(%) Median [IQR]***	***p-value***
CD3+	71.75 [64.10,79.40]	71.80 [64.10,77.00]	0.842
CD4	64.30 [50.70,75.50]	64.00 [49.30,76.30]	0.914
CD4 CD27-CD28-	3.60 [0.90,10.70]	3.00 [0.50,9.10]	0.443
CD4 CD27-CD28+	5.00 [2.90,8.50]	4.10 [2.40,7.60]	0.121
CD4 CD27+CD28-	0.50 [0.30,1.00]	0.50 [0.40,0.90]	0.311
CD4 CD27+CD28+	89.50 [79.50,94.00]	91.00 [79.80,94.60]	0.349
CD4 CM	33.05 [25.50,42.40]	33.00 [23.10,39.40]	0.188
CD4 CM CD27-CD28-	0.00 [0.00,0.10]	0.00 [0.00,0.10]	0.737
CD4 CM CD27-CD28+	3.40 [2.30,5.40]	3.10 [2.10,4.60]	0.217
CD4 CM CD27+CD28-	0.10 [0.10,0.30]	0.10 [0.00,0.40]	0.866
CD4 CM CD27+CD28+	96.30 [94.30,97.20]	96.00 [94.70,97.20]	0.905
CD4 EM	14.50 [9.30,23.50]	13.60 [6.60,21.70]	0.303
CD4 EM CD27-CD28-	16.95 [4.40,37.50]	11.60 [3.30,30.40]	0.243
CD4 EM CD27-CD28+	22.25 [16.90,29.30]	21.90 [16.40,26.10]	0.311
CD4 EM CD27+CD28-	0.40 [0.20,0.70]	0.60 [0.30,0.90]	0.015
CD4 EM CD27+CD28+	52.55 [36.90,70.50]	64.70 [39.00,72.50]	0.082
CD4 NAIVE	43.05 [29.40,55.70]	44.50 [25.60,61.50]	0.378
CD4 NAIVE CD27-CD28-	0.00 [0.00,0.00]	0.00 [0.00,0.00]	0.892
CD4 NAIVE CD27-CD28+	0.10 [0.00,0.10]	0.00 [0.00,0.10]	0.403
CD4 NAIVE CD27+CD28-	0.40 [0.20,0.80]	0.40 [0.20,0.70]	0.973
CD4 NAIVE CD27+CD28+	99.40 [98.90,99.70]	99.50 [98.90,99.70]	0.942
CD4 TEMRA	1.30 [0.60,3.60]	1.70 [0.90,3.80]	0.152
CD4 TEMRA CD27-CD28-	36.80 [10.30,68.90]	42.60 [7.50,72.20]	0.887
CD4 TEMRA CD27-CD28+	10.40 [5.70,16.80]	9.70 [5.90,15.00]	0.355
CD4 TEMRA CD27+CD28-	1.40 [0.50,2.90]	1.20 [0.40,2.80]	0.372
CD4 TEMRA CD27+CD28+	41.70 [16.20,71.70]	44.90 [16.40,74.50]	0.663
CD8+	26.20 [17.80,39.00]	27.90 [16.20,43.00]	0.772
CD8 CD27-CD28-	52.95 [31.80,67.70]	54.60 [33.80,71.40]	0.369
CD8 CD27-CD28+	3.80 [2.60,5.50]	3.40 [2.40,5.40]	0.386
CD8 CD27+CD28-	9.00 [5.70,14.50]	8.70 [5.80,16.50]	0.635
CD8 CD27+CD28+	31.05 [18.60,50.80]	27.20 [18.40,44.70]	0.262
CD8 CD45RA-CD27-	7.50 [4.20,13.80]	7.10 [4.20,13.30]	0.973
CD8 CD45RA-CD27+	17.50 [9.80,29.60]	16.10 [8.60,23.90]	0.14
CD8 CD45RA+CD27-	44.90 [27.00,60.20]	46.50 [31.10,61.20]	0.494
CD8 CD45RA+CD27+	22.00 [14.10,35.00]	22.20 [13.60,33.90]	0.966
CD8 CM	7.25 [3.80,13.70]	6.10 [3.60,10.30]	0.098
CD8 CM CD27-CD28-	0.50 [0.10,1.60]	0.60 [0.10,2.80]	0.254
CD8 CM CD27-CD28+	4.80 [2.80,6.90]	4.80 [2.80,7.50]	0.623
CD8 CM CD27+CD28-	1.00 [0.40,1.90]	1.10 [0.40,2.40]	0.411
CD8 CM CD27+CD28+	92.90 [89.00,95.20]	91.70 [87.10,93.90]	0.039
CD8 EM	19.70 [13.20,27.70]	19.90 [12.10,28.80]	0.838
CD8 EM CD27-CD28-	30.30 [12.50,51.40]	29.60 [12.10,58.60]	0.861
CD8 EM CD27-CD28+	7.50 [5.00,11.00]	8.00 [4.50,11.20]	0.747
CD8 EM CD27+CD28-	8.85 [5.60,13.80]	7.70 [4.70,16.30]	0.467
CD8 EM CD27+CD28+	47.70 [29.10,64.00]	47.70 [24.30,60.60]	0.366
CD8 NAIVE	7.30 [3.60,15.30]	8.10 [3.20,14.60]	0.929
CD8 NAIVE CD27-CD28-	0.90 [0.30,2.90]	1.20 [0.40,3.30]	0.223
CD8 NAIVE CD27-CD28+	0.20 [0.00,0.60]	0.20 [0.00,0.50]	0.786
CD8 NAIVE CD27+CD28-	5.20 [3.05,8.45]	4.70 [3.20,9.10]	0.876
CD8 NAIVE CD27+CD28+	92.60 [87.45,95.40]	91.60 [86.10,95.20]	0.299
CD8 TEMRA	53.95 [39.40,68.10]	55.40 [42.10,70.50]	0.241
CD8 TEMRA CD27-CD28-	77.10 [60.00,85.70]	77.80 [64.00,86.80]	0.6
CD8 TEMRA CD27-CD28+	2.50 [1.60,4.10]	2.40 [1.80,3.20]	0.413
CD8 TEMRA CD27+CD28-	12.05 [6.60,20.40]	11.70 [6.90,21.60]	0.952
CD8 TEMRA CD27+CD28+	6.50 [3.40,13.90]	6.30 [3.10,11.60]	0.405
CD4/CD8 RATIO	2.41 [1.29,4.23]	2.23 [1.18,4.14]	0.831
CD3+ CD4- CD8-	4.20 [2.50,7.50]	4.60 [2.60,6.80]	0.995
CD3+ CD4+ CD8+	0.80 [0.40,1.40]	0.70 [0.40,1.10]	0.073

Supplementary Table 5. Immunosenescence Parametres by CMV

	CMV Negative	CMV Positive	
	N=87	***N=510***	
	(%) Median [IQR]	***(%) Median [IQR]***	***Mann-Whitney test p-value***
CD3+	71.85 [62.30,77.50]	71.70 [64.30,79.30]	0.381
CD4	77.10 [67.80,83.70]	62.25 [49.30,73.00]	**0.000**
CD4 CD27-CD28-	0.20 [0.10,0.40]	4.95 [1.70,11.60]	**0.001**
CD4 CD27-CD28+	3.30 [2.10,4.80]	5.40 [3.00,8.90]	**0.000**
CD4 CD27+CD28-	0.75 [0.40,2.00]	0.50 [0.30,0.90]	**0.000**
CD4 CD27+CD28+	94.75 [92.20,96.50]	88.45 [77.90,93.00]	**0.000**
CD4 CM	37.55 [25.20,47.30]	32.20 [24.70,41.60]	0.437
CD4 CM CD27-CD28-	0.00 [0.00,0.00]	0.00 [0.00,0.10]	0.060
CD4 CM CD27-CD28+	3.00 [2.10,4.30]	3.45 [2.30,5.40]	**0.000**
CD4 CM CD27+CD28-	0.10 [0.10,0.40]	0.10 [0.10,0.30]	**0.004**
CD4 CM CD27+CD28+	0.10 [0.09,0.10]	96.20 [94.30,97.20]	**0.000**
CD4 EM	10.40 [7.20,14.00]	15.60 [9.60,24.70]	**0.025**
CD4 EM CD27-CD28-	0.95 [0.40,2.20]	20.60 [8.70,39.60]	0.336
CD4 EM CD27-CD28+	20.15 [15.20,25.10]	22.90 [17.60,29.30]	0.114
CD4 EM CD27+CD28-	0.65 [0.30,1.40]	0.40 [0.20,0.70]	**0.000**
CD4 EM CD27+CD28+	76.15 [69.10,82.30]	50.40 [35.10,67.00]	**0.048**
CD4 NAIVE	48.85 [36.70,63.80]	41.95 [28.00,55.30]	**0.000**
CD4 NAIVE CD27-CD28-	0.00 [0.00,0.00]	0.00 [0.00,0.10]	**0.000**
CD4 NAIVE CD27-CD28+	0.00 [0.00,0.10]	0.10 [0.00,0.10]	**0.000**
CD4 NAIVE CD27+CD28-	0.50 [0.30,1.10]	0.40 [0.20,0.80]	**0.010**
CD4 NAIVE CD27+CD28+	99.40 [98.70,99.60]	99.40 [98.90,99.70]	**0.010**
CD4 TEMRA	0.70 [0.50,1.10]	1.60 [0.80,3.90]	0.347
CD4 TEMRA CD27-CD28-	4.30 [1.80,10.00]	46.70 [18.00,72.20]	**0.001**
CD4 TEMRA CD27-CD28+	9.05 [4.30,15.30]	10.40 [6.00,16.80]	**0.002**
CD4 TEMRA CD27+CD28-	2.25 [1.00,4.80]	1.20 [0.40,2.60]	**0.000**
CD4 TEMRA CD27+CD28+	79.70 [69.30,87.80]	36.20 [13.90,64.20]	**0.000**
CD8+	15.20 [10.00,22.90]	29.30 [20.00,41.90]	**0.000**
CD8 CD27-CD28-	14.10 [7.20,32.00]	57.40 [38.00,69.30]	0.053
CD8 CD27-CD28+	3.85 [2.60,5.60]	3.70 [2.60,5.40]	**0.000**
CD8 CD27+CD28-	14.95 [7.80,22.80]	8.60 [5.50,13.40]	**0.000**
CD8 CD27+CD28+	58.20 [44.70,73.80]	27.80 [17.60,43.60]	**0.000**
CD8 CD45RA-CD27-	5.10 [2.70,9.40]	8.05 [4.60,14.60]	**0.000**
CD8 CD45RA-CD27+	31.00 [21.60,43.90]	15.40 [9.10,25.30]	0.900
CD8 CD45RA+CD27-	12.90 [6.00,25.50]	49.00 [33.00,62.00]	**0.000**
CD8 CD45RA+CD27+	37.95 [29.30,55.50]	19.50 [13.30,31.10]	**0.000**
CD8 CM	12.95 [7.50,22.50]	6.10 [3.60,11.50]	**0.000**
CD8 CM CD27-CD28-	0.20 [0.00,0.80]	0.60 [0.10,1.80]	**0.000**
CD8 CM CD27-CD28+	4.35 [2.80,6.50]	4.90 [2.80,7.10]	**0.000**
CD8 CM CD27+CD28-	0.95 [0.30,1.60]	1.00 [0.40,2.00]	**0.008**
CD8 CM CD27+CD28+	93.70 [89.60,95.50]	92.40 [88.20,95.10]	**0.000**
CD8 EM	22.80 [15.70,35.10]	19.50 [12.50,26.80]	**0.000**
CD8 EM CD27-CD28-	7.35 [4.00,14.50]	34.70 [16.00,55.70]	0.644
CD8 EM CD27-CD28+	7.65 [5.20,11.70]	7.50 [5.00,10.90]	0.088
CD8 EM CD27+CD28-	12.10 [6.80,20.80]	8.50 [5.00,13.30]	**0.000**
CD8 EM CD27+CD28+	64.00 [52.50,75.50]	43.85 [27.70,60.80]	0.462
CD8 NAIVE	14.55 [7.90,28.20]	6.45 [3.20,13.50]	**0.000**
CD8 NAIVE CD27-CD28-	0.30 [0.10,1.00]	1.10 [0.40,3.40]	**0.000**
CD8 NAIVE CD27-CD28+	0.10 [0.00,0.30]	0.20 [0.00,0.60]	**0.000**
CD8 NAIVE CD27+CD28-	6.15 [3.60,10.90]	5.05 [3.00,8.30]	0.322
CD8 NAIVE CD27+CD28+	92.90 [88.60,95.70]	92.40 [86.90,95.30]	0.112
CD8 TEMRA	32.20 [18.40,50.30]	57.05 [43.70,69.60]	0.187
CD8 TEMRA CD27-CD28-	35.80 [23.60,58.70]	79.40 [67.20,87.10]	**0.000**
CD8 TEMRA CD27-CD28+	2.60 [1.60,3.90]	2.50 [1.60,3.90]	**0.010**
CD8 TEMRA CD27+CD28-	31.10 [18.80,41.00]	10.70 [5.80,17.60]	**0.000**
CD8 TEMRA CD27+CD28+	22.95 [11.60,36.60]	5.80 [3.10,11.30]	**0.000**
CD4/CD8 RATIO	5.03 [3.03,7.95]	2.18 [1.18,3.62]	**0.000**
CD3+ CD4- CD8-	3.60 [2.30,7.60]	4.30 [2.60,7.40]	0.979
CD3+ CD4+ CD8+	0.65 [0.30,1.10]	0.80 [0.50,1.50]	**0.000**

Supplementary Table 6. Immunosenescence Parametres by CMV and Gender

	Male CMV Negative *N=35* ***(%) Median [IQR]***	Female CMV Negative *N=52* ***(%) Median [IQR]***	***Mann-Whitney test p-value***	Male CMV Positive *N=190* ***(%) Median [IQR]***	Female CMV Positive *N=320* ***(%) Median [IQR]***	***Mann-Whitney test p-value***
CD3+	67.30 [58.90,77.50]	73.10 [62.45,77.75]	0.432	70.60 [63.60,78.30]	72.50 [64.70,79.40]	0.119
CD4	76.10 [66.70,81.70]	77.30 [70.10,86.15]	0.212	57.70 [46.10,69.10]	64.10 [52.10,75.50]	**0.000**
CD4 CD27-CD28-	0.15 [0.10,0.80]	0.20 [0.10,0.40]	0.563	6.30 [1.80,13.50]	4.10 [1.70,10.90]	0.127
CD4 CD27-CD28+	3.85 [2.80,5.60]	2.45 [1.70,4.50]	**0.006**	6.20 [3.70,10.50]	4.80 [2.90,7.90]	**0.001**
CD4 CD27+CD28-	0.65 [0.30,1.30]	0.90 [0.50,2.20]	0.105	0.50 [0.30,0.90]	0.50 [0.30,0.90]	0.229
CD4 CD27+CD28+	94.30 [89.00,95.90]	94.90 [92.40,97.05]	0.142	85.40 [72.70,92.50]	89.10 [80.00,93.30]	**0.004**
CD4 CM	40.55 [33.10,51.60]	34.40 [23.55,41.65]	**0.017**	35.20 [27.70,43.20]	31.30 [23.20,40.40]	**0.005**
CD4 CM CD27-CD28-	0.00 [0.00,0.00]	0.00 [0.00,0.00]	**0.005**	0.00 [0.00,0.10]	0.00 [0.00,0.10]	**0.006**
CD4 CM CD27-CD28+	3.30 [2.40,5.40]	2.70 [1.85,4.10]	**0.005**	3.70 [2.40,5.70]	3.30 [2.30,4.70]	**0.000**
CD4 CM CD27+CD28-	0.10 [0.10,0.20]	0.25 [0.10,0.80]	0.061	0.10 [0.00,0.30]	0.10 [0.10,0.30]	0.944
CD4 CM CD27+CD28+	96.45 [94.60,97.20]	96.70 [95.25,97.35]	0.121	96.00 [93.60,97.00]	96.40 [94.90,97.30]	0.316
CD4 EM	12.60 [8.20,19.90]	8.95 [6.00,12.30]	0.139	16.90 [10.50,28.60]	14.50 [9.30,22.80]	0.082
CD4 EM CD27-CD28-	0.95 [0.50,2.20]	0.95 [0.35,2.35]	**0.043**	20.40 [9.00,42.10]	20.80 [7.80,38.60]	0.163
CD4 EM CD27-CD28+	22.25 [15.20,30.40]	19.90 [15.15,22.55]	0.597	24.30 [18.60,30.30]	22.00 [16.50,28.40]	**0.019**
CD4 EM CD27+CD28-	0.60 [0.30,1.00]	0.90 [0.30,1.55]	0.324	0.40 [0.20,0.60]	0.40 [0.20,0.70]	0.437
CD4 EM CD27+CD28+	74.70 [53.10,81.60]	77.25 [72.70,82.55]	0.189	48.30 [31.20,64.40]	51.40 [37.90,68.30]	**0.013**
CD4 NAIVE	43.60 [23.60,52.90]	53.00 [39.35,66.40]	0.237	36.90 [21.90,47.70]	45.50 [32.10,57.60]	0.059
CD4 NAIVE CD27-CD28-	0.00 [0.00,0.00]	0.00 [0.00,0.00]	**0.047**	0.00 [0.00,0.10]	0.00 [0.00,0.00]	**0.022**
CD4 NAIVE CD27-CD28+	0.00 [0.00,0.10]	0.00 [0.00,0.10]	0.828	0.10 [0.00,0.20]	0.00 [0.00,0.10]	**0.002**
CD4 NAIVE CD27+CD28-	0.55 [0.20,0.80]	0.50 [0.30,1.55]	0.084	0.40 [0.20,0.90]	0.40 [0.20,0.70]	**0.030**
CD4 NAIVE CD27+CD28+	99.35 [99.00,99.60]	99.40 [98.45,99.60]	0.407	99.40 [98.80,99.70]	99.40 [98.90,99.70]	0.783
CD4 TEMRA	0.80 [0.60,1.30]	0.60 [0.40,1.00]	0.571	1.70 [0.70,3.90]	1.60 [0.80,3.90]	0.292
CD4 TEMRA CD27-CD28-	3.85 [1.80,15.00]	4.50 [1.70,9.80]	0.758	46.75 [15.10,74.50]	46.00 [18.40,71.20]	0.982
CD4 TEMRA CD27-CD28+	7.90 [4.00,15.90]	9.15 [4.55,14.45]	0.869	11.50 [6.70,18.20]	10.00 [5.50,16.10]	**0.042**
CD4 TEMRA CD27+CD28-	1.10 [0.70,3.60]	2.85 [1.80,5.35]	**0.018**	1.20 [0.30,2.60]	1.20 [0.50,2.60]	0.436
CD4 TEMRA CD27+CD28+	81.10 [63.30,89.50]	79.70 [71.55,86.95]	0.984	36.75 [10.30,63.10]	35.80 [16.10,64.60]	0.292
CD8+	17.90 [12.50,23.10]	15.00 [9.90,22.05]	0.256	32.60 [22.50,44.90]	26.50 [18.00,38.20]	**0.000**
CD8 CD27-CD28-	13.80 [8.40,33.60]	14.10 [6.80,30.50]	0.659	59.60 [41.20,72.80]	54.90 [36.90,68.00]	**0.029**
CD8 CD27-CD28+	5.00 [4.00,6.60]	3.00 [2.05,4.75]	**0.001**	4.10 [2.80,5.80]	3.60 [2.50,5.00]	**0.018**
CD8 CD27+CD28-	14.60 [6.90,22.80]	15.40 [11.00,23.00]	0.400	8.90 [4.90,14.30]	8.40 [5.70,12.70]	0.480
CD8 CD27+CD28+	56.20 [40.20,71.80]	60.15 [47.90,76.05]	0.518	24.20 [15.50,38.20]	29.40 [19.90,47.70]	**0.001**
CD8 CD45RA-CD27-	6.25 [4.50,9.80]	4.10 [2.30,7.85]	**0.019**	9.90 [5.20,18.20]	7.20 [4.20,12.40]	**0.001**
CD8 CD45RA-CD27+	37.75 [16.80,51.20]	29.25 [22.35,39.80]	0.432	15.40 [8.80,25.30]	15.40 [9.10,24.50]	0.814
CD8 CD45RA+CD27-	14.05 [6.30,33.20]	12.50 [5.90,24.05]	0.548	50.60 [34.20,62.80]	48.70 [32.50,61.70]	0.485
CD8 CD45RA+CD27+	32.80 [25.00,47.00]	43.05 [32.45,60.35]	**0.031**	16.50 [10.50,27.40]	22.00 [14.50,33.70]	**0.000**
CD8 CM	15.95 [7.00,25.10]	11.75 [8.15,19.20]	0.453	5.80 [3.30,10.80]	6.40 [3.80,11.60]	0.190
CD8 CM CD27-CD28-	0.40 [0.00,1.60]	0.20 [0.00,0.50]	0.224	0.80 [0.10,2.10]	0.50 [0.10,1.70]	**0.002**
CD8 CM CD27-CD28+	6.20 [4.30,7.90]	3.60 [2.30,5.15]	0.099	5.75 [3.50,8.40]	4.40 [2.50,6.60]	**0.000**
CD8 CM CD27+CD28-	0.75 [0.20,1.50]	1.20 [0.50,1.85]	0.292	1.00 [0.30,2.20]	1.00 [0.40,1.90]	0.082
CD8 CM CD27+CD28+	91.00 [88.60,94.00]	94.80 [91.65,96.15]	**0.000**	91.70 [87.20,93.90]	93.30 [89.40,95.50]	**0.000**
CD8 EM	25.20 [18.10,36.00]	21.85 [14.00,32.15]	0.278	21.50 [14.60,31.00]	18.40 [11.70,25.30]	0.970
CD8 EM CD27-CD28-	6.30 [4.60,12.50]	8.65 [3.70,15.40]	**0.005**	35.30 [16.50,58.70]	33.90 [15.30,53.10]	**0.000**
CD8 EM CD27-CD28+	9.20 [7.20,13.30]	6.55 [4.90,10.60]	0.696	7.90 [4.70,11.40]	7.20 [5.00,10.70]	0.181
CD8 EM CD27+CD28-	9.35 [5.70,20.30]	13.45 [8.20,22.05]	**0.033**	8.10 [4.70,13.40]	8.80 [5.30,13.20]	0.312
CD8 EM CD27+CD28+	60.50 [52.50,75.40]	65.40 [52.50,76.85]	0.178	43.00 [21.30,57.40]	45.70 [28.60,61.80]	0.361
CD8 NAIVE	12.65 [6.20,21.50]	15.85 [8.75,36.50]	0.586	4.90 [1.80,9.40]	7.60 [4.00,16.60]	**0.032**
CD8 NAIVE CD27-CD28-	0.55 [0.20,1.10]	0.20 [0.05,0.70]	0.128	1.20 [0.40,4.70]	0.90 [0.30,2.90]	0.056
CD8 NAIVE CD27-CD28+	0.20 [0.00,0.40]	0.10 [0.00,0.20]	0.095	0.30 [0.00,0.80]	0.20 [0.00,0.50]	0.071
CD8 NAIVE CD27+CD28-	5.05 [3.60,8.30]	6.90 [3.50,11.65]	0.307	5.20 [3.20,9.50]	4.90 [2.80,7.60]	0.101
CD8 NAIVE CD27+CD28+	93.35 [89.30,95.70]	92.70 [88.00,95.45]	0.666	90.80 [85.50,94.90]	93.00 [87.70,95.50]	**0.024**
CD8 TEMRA	35.00 [18.40,58.50]	30.95 [18.35,48.05]	0.498	57.20 [45.70,69.40]	57.00 [42.10,69.60]	0.229
CD8 TEMRA CD27-CD28-	43.25 [24.10,58.70]	33.25 [23.25,58.95]	0.485	80.70 [66.70,88.40]	79.10 [67.50,86.50]	0.232
CD8 TEMRA CD27-CD28+	2.80 [2.00,4.60]	2.40 [1.50,3.90]	0.327	2.50 [1.60,3.80]	2.50 [1.70,4.10]	0.452
CD8 TEMRA CD27+CD28-	25.90 [16.30,37.90]	31.70 [20.15,43.65]	0.372	10.70 [5.20,18.50]	10.70 [6.20,17.20]	0.988
CD8 TEMRA CD27+CD28+	21.75 [12.00,32.40]	23.35 [11.40,36.95]	0.762	4.90 [2.70,9.30]	6.20 [3.30,12.10]	**0.011**
CD4/CD8 RATIO	4.16 [2.70,6.82]	5.19 [3.25,8.79]	0.222	1.77 [1.03,3.02]	2.41 [1.38,4.06]	**0.000**
CD3+ CD4- CD8-	5.10 [2.30,9.00]	3.25 [2.20,6.40]	0.250	4.30 [2.60,7.60]	4.30 [2.50,7.30]	0.366
CD3+ CD4+ CD8+	0.55 [0.30,1.10]	0.80 [0.30,1.15]	0.533	0.60 [0.30,1.30]	0.80 [0.50,1.60]	**0.002**

Supplementary Table 7. Cox Regression Models by Biomarkers Tertiles. Cardiovascular Mortality (CVD) vs Non-Cardiovascular Mortality (Non-CVD).

CVD Mortality Cox Regression Model : CD8 TEMRA

		Nº Individuals	Nº Events	Unadjusted	p	Adjusted[1]	p
Lymphocytes (x 10^9/l)	<= 1.42	126	76	1	**0.031**	1	0.075
	1.43 - 1.93	143	78	0.84 (0.58 - 1.20)		0.86 (0.60 - 1.24)	
	1.94+	137	61	0.60 (0.41 - 0.88)		0.64 (0.43 - 0.95)	
Basophils (x 10^9/l)	<= .03	189	91	1	**0.018**	1	**0.031**
	.04 - .05	128	74	1.65 (1.17 - 2.32)		1.60 (1.13 - 2.28)	
	.06+	89	50	1.21 (0.81 - 1.80)		1.20 (0.80 - 1.79)	
TGF-beta (ng/ml)	<= 13.17	138	86	1	**0.002**	1	**0.038**
	13.18 - 17.36	125	55	0.51 (0.36 - 0.74)		0.62 (0.43 - 0.90)	
	17.37+	126	69	0.79 (0.56 - 1.14)		0.87 (0.60 - 1.25)	
Haemoglobin (g/dl)	<= 12.30	115	71	1	0.393	1	0.962
	12.31 - 13.60	152	75	0.85 (0.59 - 1.21)		0.96 (0.67 - 1.38)	
	13.61+	139	69	0.78 (0.54 - 1.12)		0.95 (0.66 - 1.38)	
**CD8 TEMRA (%)**	<= 45.20	135	60	1	**0.029**	1	0.141
	45.21 - 63.80	126	72	1.49 (1.03 - 2.14)		1.42 (0.98 - 2.06)	
	63.81+	122	72	1.60 (1.11 - 2.31)		1.37 (0.94 - 2.01)	

CVD Mortality Cox Regression Model : CD8 CD27-CD28+

		Nº Individuals	Nº Events	Unadjusted	p	Adjusted[1]	p
Lymphocytes (x 10^9/l)	<= 1.42	126	76	1	0.145	1	0.164
	1.43 - 1.93	143	78	0.92 (0.64 - 1.31)		0.94 (0.65 - 1.36)	
	1.94+	137	61	0.69 (0.47 - 1.02)		0.70 (0.47 - 1.04)	
Basophils (x 10^9/l)	<= .03	189	91	1	**0.023**	1	**0.031**
	.04 - .05	128	74	1.61 (1.15 - 2.28)		1.60 (1.13 - 2.27)	
	.06+	89	50	1.29 (0.87 - 1.91)		1.27 (0.85 - 1.90)	
TGF-beta (ng/ml)	<= 13.17	138	86	1	**0.002**	1	0.055
	13.18 - 17.36	125	55	0.52 (0.36 - 0.75)		0.64 (0.44 - 0.92)	
	17.37+	126	69	0.77 (0.54 - 1.10)		0.86 (0.59 - 1.23)	
Haemoglobin (g/dl)	<= 12.30	115	71	1	0.288	1	0.944
	12.31 - 13.60	152	75	0.81 (0.57 - 1.15)		0.96 (0.67 - 1.37)	
	13.61+	139	69	0.76 (0.53 - 1.09)		0.94 (0.65 - 1.37)	
**CD8 CD27-CD28+ (%)**	<= 1.90	128	78	1	0.051	1	**0.029**
	1.91 - 3.40	129	64	0.70 (0.49 - 1.00)		0.68 (0.47 - 0.97)	
	3.41+	126	62	0.68 (0.47 - 0.96)		0.65 (0.45 - 0.93)	

Non-CVD Mortality Cox Regression Model : CD8 TEMRA

		Nº Individuals	Nº Events	Unadjusted	p	Adjusted[1]	p
Lymphocytes (x 10^9/l)	<= 1.42	120	70	1	**0.037**	1	0.255
	1.43 - 1.93	121	56	0.67 (0.45 - 1.00)		0.77 (0.52 - 1.15)	
	1.94+	132	56	0.61 (0.41 - 0.91)		0.73 (0.49 - 1.09)	
Basophils (x 10^9/l)	<= .03	190	92	1	0.930	1	0.809
	.04 - .05	106	52	1.06 (0.73 - 1.55)		1.11 (0.75 - 1.63)	
	.06+	77	38	0.98 (0.65 - 1.50)		0.96 (0.63 - 1.46)	
TGF-beta (ng/ml)	<= 13.17	102	50	1	0.574	1	0.770
	13.18 - 17.36	135	65	0.97 (0.64 - 1.45)		1.03 (0.69 - 1.56)	
	17.37+	119	62	1.17 (0.76 - 1.79)		1.15 (0.75 - 1.77)	
Haemoglobin (g/dl)	<= 12.30	126	82	1	**0.000**	1	**0.003**
	12.31 - 13.60	121	44	0.43 (0.29 - 0.64)		0.53 (0.35 - 0.79)	
	13.61+	126	56	0.51 (0.36 - 0.75)		0.60 (0.41 - 0.89)	
**CD8 TEMRA (%)**	<= 45.20	126	51	1	**0.031**	1	**0.030**
	45.21 - 63.80	114	60	1.40 (0.94 - 2.07)		1.42 (0.95 - 2.12)	
	63.81+	112	62	1.69 (1.14 - 2.51)		1.70 (1.14 - 2.54)	

Non-CVD Mortality Cox Regression Model : CD8 CD27-CD28+

		Nº Individuals	Nº Events	Unadjusted	p	Adjusted[1]	p
Lymphocytes (x 10^9/l)	<= 1.42	120	70	1	0.214	1	0.796
	1.43 - 1.93	121	56	0.78 (0.53 - 1.14)		0.94 (0.63 - 1.40)	
	1.94+	132	56	0.71 (0.48 - 1.06)		0.87 (0.58 - 1.30)	
Basophils (x 10^9/l)	<= .03	190	92	1	0.909	1	0.716
	.04 - .05	106	52	1.08 (0.74 - 1.58)		1.16 (0.79 - 1.71)	
	.06+	77	38	1.01 (0.66 - 1.53)		0.99 (0.65 - 1.51)	
TGF-beta (ng/ml)	<= 13.17	102	50	1	0.662	1	0.926
	13.18 - 17.36	135	65	0.97 (0.65 - 1.46)		1.02 (0.68 - 1.53)	
	17.37+	119	62	1.14 (0.75 - 1.75)		1.08 (0.71 - 1.65)	
Haemoglobin (g/dl)	<= 12.30	126	82	1	**0.000**	1	**0.001**
	12.31 - 13.60	121	44	0.40 (0.27 - 0.59)		0.49 (0.33 - 0.74)	
	13.61+	126	56	0.49 (0.34 - 0.70)		0.58 (0.40 - 0.85)	
**CD8 CD27-CD28+ (%)**	<= 1.90	112	62	1	0.139	1	**0.037**
	1.91 - 3.40	123	58	0.85 (0.58 - 1.24)		0.76 (0.51 - 1.11)	
	3.41+	117	53	0.67 (0.46 - 1.00)		0.59 (0.39 - 0.88)	

Values are expressed as Mean (Lower, Upper bound of 95%CI). [1]Adjusted for Tertiles of pro BNP, Cancer and Frailty

Supplementary Figure 1. Phenotype of CD8 T cells in young (<55y) and elderly (>86y) participants.

A. KLRG1 expression in $CD8^+$ subpopulations

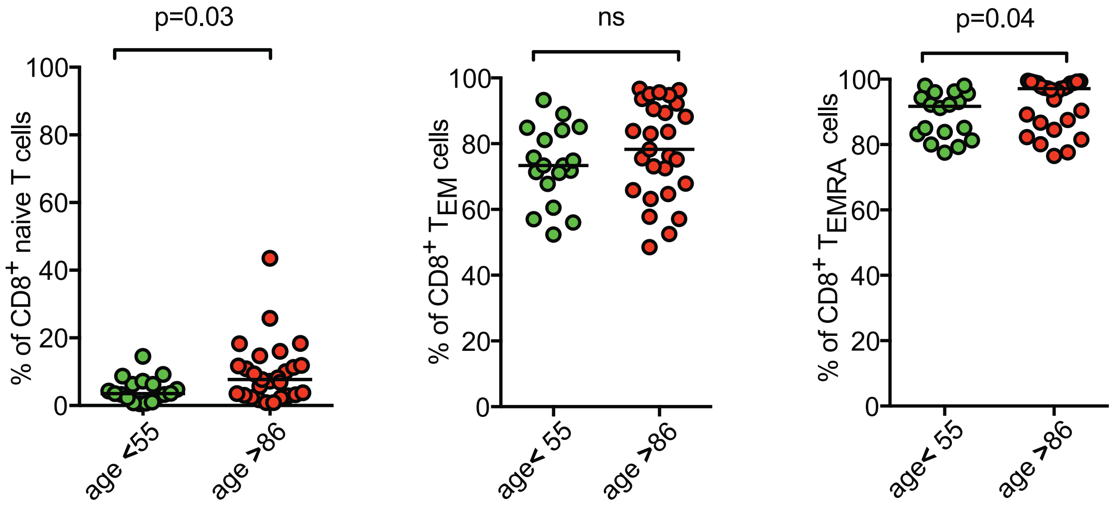

B. Distribution of CD27/CD28 subpopulations in $CD8^+$ TEMRA cells

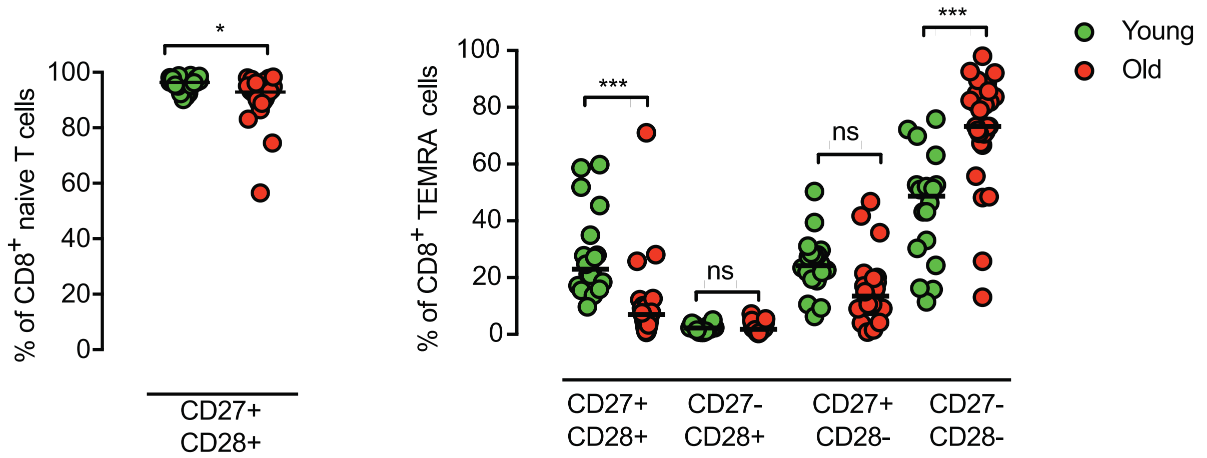

C. KLRG1 expression in $CD8^+$ TEMRA subpopulations

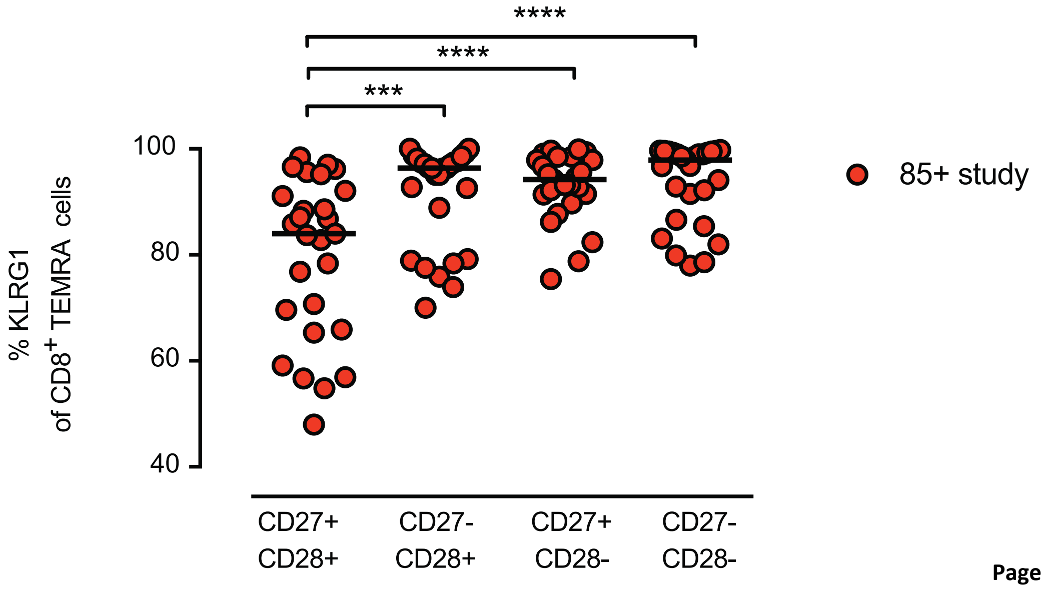

Supplementary Figure 2: Mouse flow-cytometry gating scheme

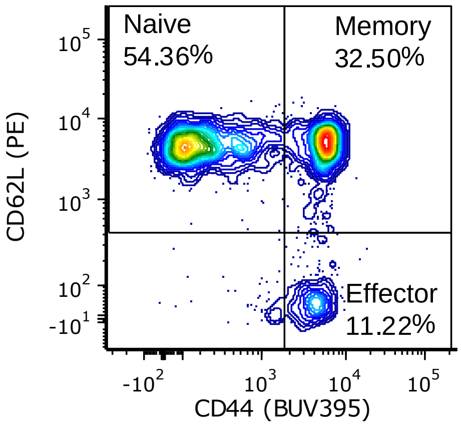

Exp65 - NAV treated old mice #3
file B5, mouse B13

Supplementary Figure S3. Flowchart on covariates selection

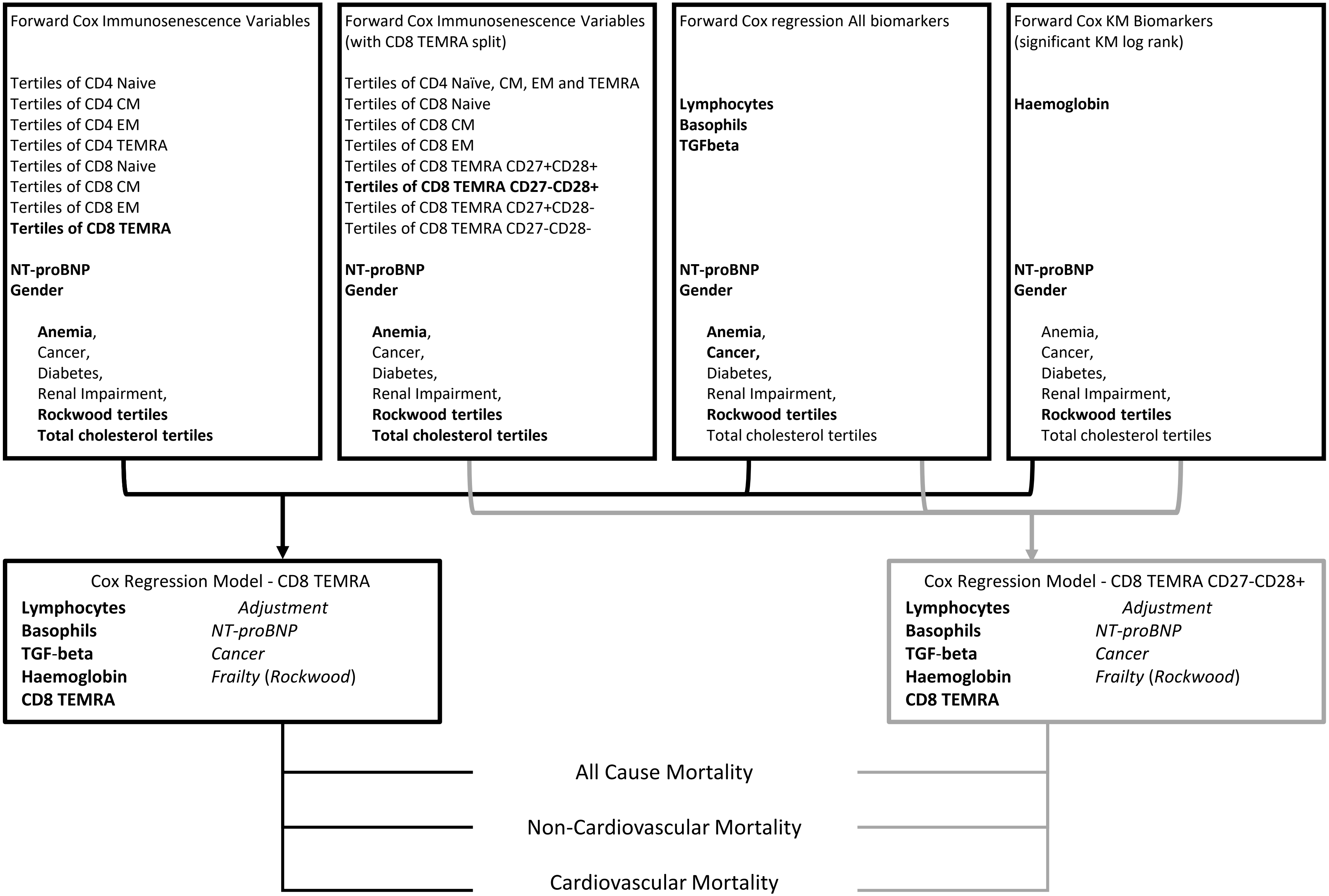

Supplement: Supplementary file 1 — Supplementary Material [file 41514_2019_41_MOESM1_ESM.pdf]
